# An Unusual Mechanism of Closure of Muscular Ventricular Septal Defects

**DOI:** 10.1155/2017/4303298

**Published:** 2017-10-10

**Authors:** Soham Dasgupta, Ashraf M. Aly

**Affiliations:** ^1^Department of Pediatrics, University of Texas Medical Branch, Galveston, TX 77555, USA; ^2^Department of Pediatric Cardiology, University of Texas Medical Branch, Galveston, TX 77555, USA

## Abstract

Ventricular septal defects (VSDs) are the most common congenital heart defects. Most of the small or moderate size (<6 mm) muscular VSDs close spontaneously within the first two years of life. The usual mechanism of spontaneous closure involves muscular tissue encroachment with superimposed fibrosis or primary fibrous tissue formation around the margins of the defect. We describe an unusual mechanism of spontaneous closure of a muscular VSD.

## 1. Introduction

Ventricular septal defects are the most common congenital heart defects (1) and are classified by location into muscular, membranous, supracristal, or inlet. Spontaneous closure of a VSD depends on multiple factors including site, size, and age of the patient. The mechanism of closure depends on the location of the VSD. We describe a case of an unusual mechanism of spontaneous closure of a muscular VSD.

## 2. Case Report

**Figure 1:**
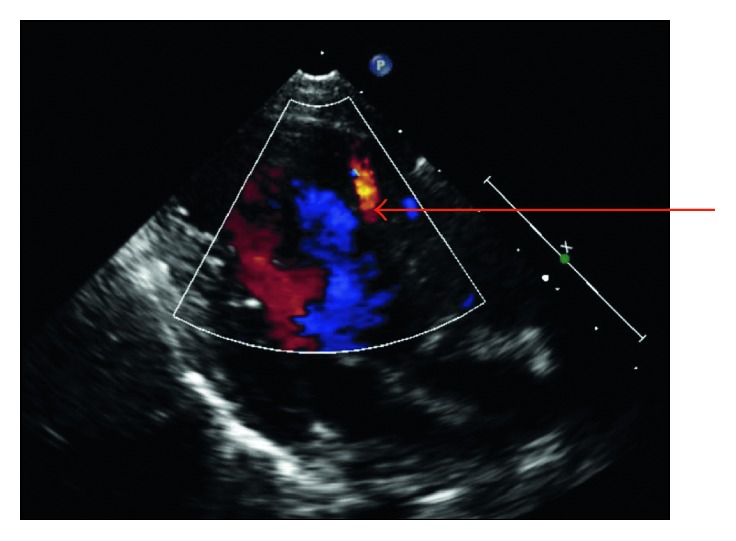
Parasternal long-axis view demonstrating a laminar flow from the LV to the interventricular septum (red arrow).

**Figure 3:**
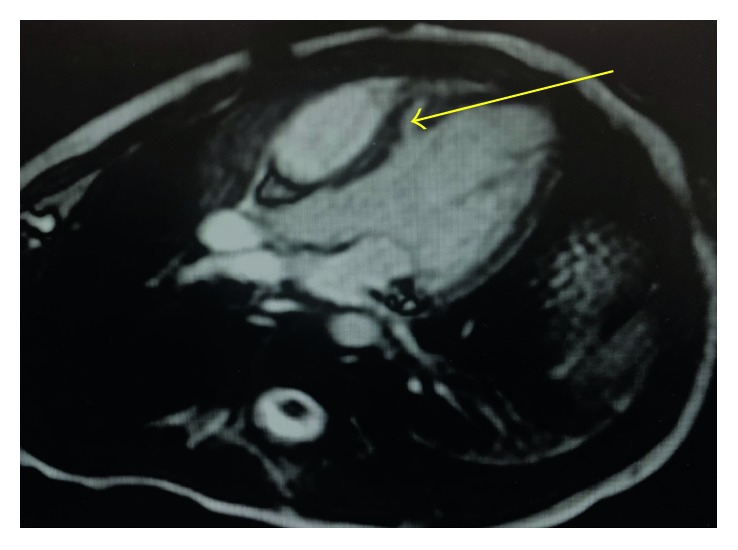
Cardiac MRI (multislice 4-chamber view) showing a small muscular VSD (yellow arrow).

A two-month-old healthy female infant was referred to pediatric cardiology for evaluation of a heart murmur. Except for a grade 3/6 holosystolic murmur, the rest of the cardiac examination was unremarkable. An echocardiogram (ECHO) showed a 4 mm midmuscular VSD with a left-to-right shunt. The patient remained asymptomatic but did not follow up with cardiology for three years. Cardiac examination was unremarkable, and the murmur was no longer appreciated. A repeat ECHO revealed a 4 mm midmuscular VSD measured from the left ventricular (LV) side. The color Doppler showed two laminar flows with opposing directions across the interventricular septum (IVS). There was no left-to-right shunt communicating with the right ventricular (RV) cavity. Close observation of the color Doppler showed a continuous loop starting from the LV, across the VSD continuing through the RV trabeculations, and back to the LV without ever communicating with the RV cavity (Figures 1() and 2()). A cardiac magnetic resonance imaging (MRI) confirmed that postulation and showed the small muscular VSD tract covered by hypertrophied trabeculations on the RV side (Figures 3() and 4()). The MRI also demonstrated normal biventricular systolic function and a pulmonary to the systemic blood flow (Q_p_ : Q_s_) ratio of 1 : 1.

## 3. Discussion

VSDs can be isolated or associated with other congenital heart defects [1]. The incidence of VSDs ranges between 1.5 and 4.2 cases for every 1000 live term infants [2]. They are more common in premature infants, with an incidence of 4.5–7 cases for every 1000 live births. Membranous VSD is the most common type of VSD (60–70%). Muscular VSD is the second most common type, accounting for as many as 20–30% of cases identified in most surgical or autopsy series.

The spontaneous closure of VSDs depends on the age/gender of the patient and the size and site of the defect [1]. Most of the VSDs close spontaneously within the first two years of life [3, 4]. Afterwards, the chance of spontaneous closure diminishes remarkably. Muscular and membranous VSDs with diameters less than 6 mm have the best chance of spontaneous closure [5, 6]. Supracristal and inlet VSDs usually do not close spontaneously.

Several studies have discussed the different mechanisms of VSD closure. Membranous VSDs close by adherence of tricuspid valve leaflets, creating an aneurysm that closes the defect [1, 7, 8]. It has been suggested that the spontaneous closure of muscular VSDs could be due to muscular encroachment of the septal defect along with superimposed fibrosis or by fibrous tissue formation around the margins leading to apposition of the edges of the defect [9].

We describe a case of an unusual mechanism of spontaneous closure of a midmuscular VSD. The VSD in our patient appears to have closed from the RV side secondary to trabecular hypertrophy but remains open from the LV side. This leads to an unusual flow pattern which could initially be misdiagnosed as bidirectional shunting by the color Doppler ECHO. However, the laminar nature of the flow, lack of its communication with the RV cavity, and the absence of symptoms made the possibility of a bidirectional shunt unlikely. Hence, a cardiac MRI was obtained to better delineate the anatomy. Based on the color Doppler and MRI images, we assume that the flow forms a continuous loop starting from the LV, through the VSD and the RV trabeculations, and back to the LV without ever communicating with the RV cavity. Though rare, it is important to be aware of this unusual method of closure of muscular VSDs which will prevent unnecessary investigations and anxiety.

## 4. Conclusion

Multiple mechanisms have been proposed for the closure of muscular VSDs, and almost all of them include formation of fibrous tissue leading to closure of the defect. This case highlights an unusual method of spontaneous closure of a muscular VSD leading to an unusual color Doppler flow. This flow pattern could be mistaken for bidirectional shunting. However, its laminar nature and lack of communication with the RV cavity make this possibility unlikely.

## Figures and Tables

**Figure 2:**
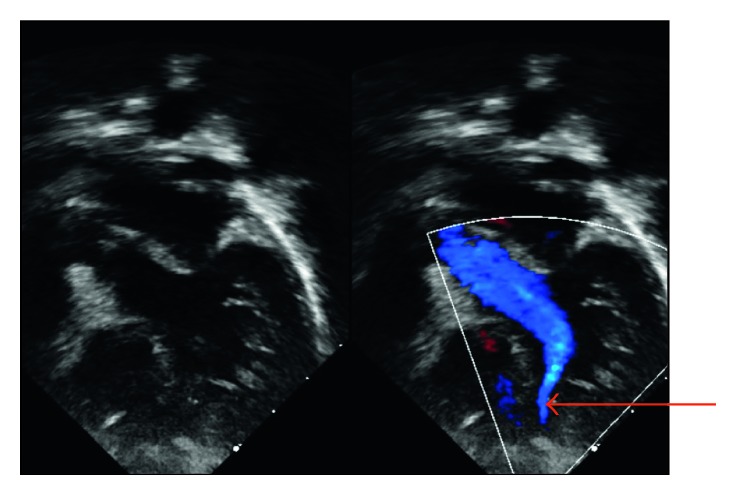
Four-chamber view showing a laminar flow returning to the LV from the interventricular septum (red arrow).

**Figure 4:**
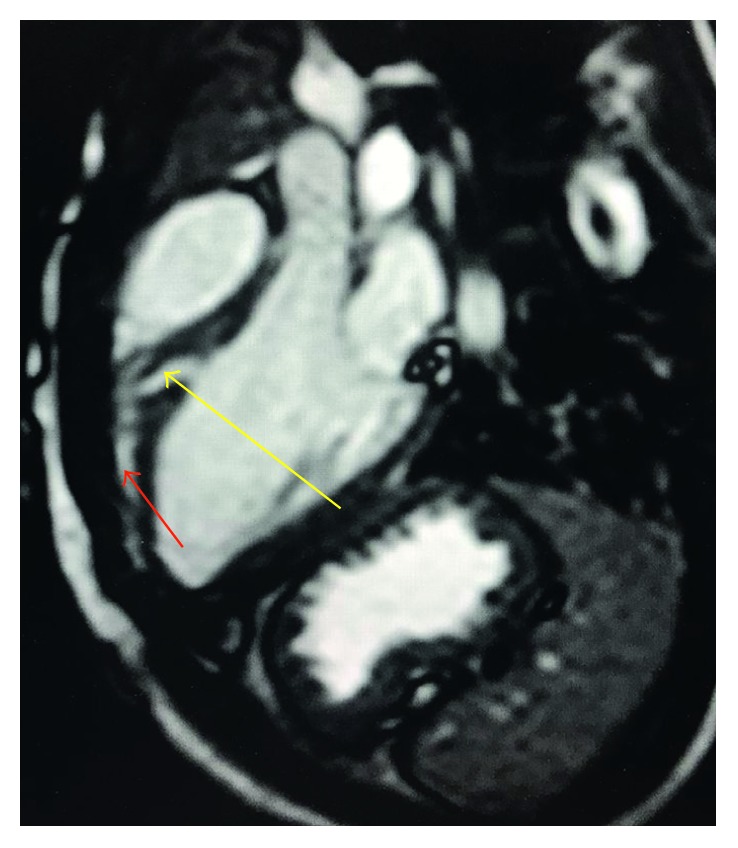
Cardiac MRI (left ventricular outflow tract view) showing a small muscular VSD (yellow arrow) covered by hypertrophied trabeculations on the RV side (red arrow).
